# Sensing Magnetic Fields with Magnetosensitive Ion Channels

**DOI:** 10.3390/s18030728

**Published:** 2018-02-28

**Authors:** Igor Goychuk

**Affiliations:** Institute of Physics and Astronomy, University of Potsdam, Karl-Liebknecht-Str. 24/25, 14476 Potsdam-Golm, Germany; igoychuk@uni-potsdam.de

**Keywords:** magnetic nanoparticles, ion channels, viscoelastic effects and anomalous diffusion, non-exponential statistics, influence of weak magnetic fields on living systems

## Abstract

Magnetic nanoparticles are met across many biological species ranging from magnetosensitive bacteria, fishes, bees, bats, rats, birds, to humans. They can be both of biogenetic origin and due to environmental contamination, being either in paramagnetic or ferromagnetic state. The energy of such naturally occurring single-domain magnetic nanoparticles can reach up to 10–20 room $k_{B}T$ in the magnetic field of the Earth, which naturally led to supposition that they can serve as sensory elements in various animals. This work explores within a stochastic modeling framework a fascinating hypothesis of magnetosensitive ion channels with magnetic nanoparticles serving as sensory elements, especially, how realistic it is given a highly dissipative viscoelastic interior of living cells and typical sizes of nanoparticles possibly involved.

## 1. Introduction

Influence of weak electromagnetic fields on living species is perceived by many scientists as a controversial subject matter. Nevertheless, there is a huge body of evidence of a substantial impact ([1,2,3,4], see, especially, the book by Binhi [5] and the references therein). One of such manifestations is given by the microwave auditory effect or Allan Frey hearing effect [6,7,8,9,10,11,12,13,14,15,16], an auditory perception of microwave pulses by humans and animals, which earlier has been considered mysterious. Now, the mystery of this effect is completely resolved within a thermoelastic theory [7,8,9,10,11,12,13,14,15,16] of acoustic wave production in closed resonators (e.g., human or animal head) filled with microwave absorbing tissues having a very large water content (think about heating of food in microwave oven, to realize a possible physical reason). Good reviews are available [13,14,15] and the theory and experiment agree convincingly well. The energy absorption per pulse of 16μJ/kg sufficient to produce the microwave hearing effect [9,13] in humans is 36,000 times lower that the maximal limit of 576 J/kg permitted in the IEEE C95.1 radiation safety standard [13], and a corresponding pulse-like elevation of temperature is really tiny, about $10^{-6\;\circ }C$ per pulse [13,15], however, rapid (about μs). This is currently probably the only one of known profound effects of weak electromagnetic fields on living systems which is explained completely. However, a direct influence of GHz and THz waves on neuronal tissues, which is also evidenced experimentally, is still not convincingly explained and this is the subject of ongoing research [17,18]. Epidemiological evidence for follow-up health effects including a spectrum of neuropsychiatric disorders is extensive, see e.g., in [19], and the references therein. Sensing and navigation of various living species such as magnetosensitive bacteria, fishes, turtles, bees, bats, rats, birds, etc. in the weak magnetic field of the Earth (about 50 μT) presents another well established effect [5,20,21]. Differently from electric fields, quasi-static magnetic fields are practically not screened by moving ions and counter-ions, and can deeply penetrate into biological tissues [5]. Currently, such a high sensitivity to magnetic fields presents a puzzle with two basic hypothetical theories proposed to resolve it. One suggests that this is a non-thermal quantum effect based on spin-dependent electron transfer reactions [20,22,23,24] in certain proteins related to vision [21,23,24,25] and it is light-dependent. Whereas it might indeed be relevant for certain birds, it certainly cannot be applied to animals navigating in dark such as fishes, or nocturnal mammals such as bats. An alternative theory is based on a wide presence of magnetic nanoparticles (MNPs), such as magnetite (iron oxide, ${\mathrm{Fe}}_{3}O_{4}$), in organs and tissues of many biological species [26,27,28,29], including human brain [30,31,32,33,34]. The biomagnetite nanoparticles were first discovered in magnetotactic bacteria [35], where they form chains of magnetosomes [35,36,37]. Whereas magnetite nanoparticles produced by, e.g., diesel engines, have typically a rounded form, the biomagnetite particles have typically a prismatic or elongated parallelepiped form [26,27]. This characteristic feature is even used as a plausible criterion for that magnetite nanoparticles found in a Martian meteorite ALH84001 may possibly evidence for the existence of life on this planet in the past [38]. The presence of biomagnetite is currently accepted by NASA as one of possible traces of life. How big can such particles be? For example, a typical volume size corresponding to the maximum of probability distribution of biomagnetite particles in a bacterial strain named NIc is $V=144\times 106\times 106\;{\mathrm{nm}}^{3}$ [37]. Crystals of such size are in a single-domain ferrimagnetic state at room temperatures [26,27] with saturation magnetization $M_{s}=4.8\cdot 10^{5}$ A/m. Hence, such particles have a permanent magnetic dipole moment $\mu =M_{s}V=7.28\times 10^{-16}\;A\cdot m^{2}$ and their magnetic energy in the magnetic field of the Earth is $E_{B}=\mu B_{e}\approx 3.88\times 10^{-20}\;J∼$9.47 $k_{B}T_{r}$, i.e., almost 10 room $k_{B}T$, well over a typical thermal energy. Interestingly, about 10% of biomagnetite particles in human brain have sizes corresponding to such a large magnetic energy [30] with overall particles density 4 ng per gram of tissue in the gray matter on average, or about $10^{6}$ particles per gram of tissue. In hippocampus, their density is even larger, 50 ng/g [32]. A recent PNAS paper [33] questioned the biological origin of this magnetite. Indeed, most particles found in the polluted brains of deceased persons who lived in Mexico City and Manchester were of anthropogenic origin [34]. They were of a rounded form with a median diameter of 18 nm. However, their specific density ranged from 0.2 to 12 μg/g of dry tissue [33], i.e., it was from 50 to 3000 times larger than one in unpolluted human brains [30]. No wonder that biomagnetite concentration is such brains was negligible with respect to one incurred by the environmental pollution. As a side remark, a brain polluted by magnetite nanoparticles will absorb more incident microwave energy than one free of such particles, or having a natural concentration. In particular, the local temperature around such particles can be increased stronger. A standard argumentation [39,40] that a temperature increase by $10^{-4}-10^{-5\;\circ }C$ is physically and biologically irrelevant is wrong, as evidenced by the theory and practice of microwave auditory effect [13,15]. This is because the temperature increase is very rapid at a pulse-modulation. Magnetic nanoparticles are known to heavily absorb microwaves between 0.5 and 10.0 GHz through ferromagnetic resonance and also produce hypersound via magnetoacoustic effect [41,42]. The physics of this effect is even used to thermally destroy cancer cells, which are known to accumulate magnetic nanoparticles [43], by using sufficiently strong microwaves (magnetic hyperthermia). An excess accumulation of magnetic nanoparticles in biological tissues can generally be a signature of decease.

Currently, magnetic control of cellular processes using biofunctional MNPs presents a hot topic (see a recent review [44] and the references therein). Apart from hyperthermia-based therapy and controlled drug delivery [43], this includes, e.g., genetically targeted control of neuronal system [45], control of calcium influx in cortical neuronal networks [46], and control of inner ear hair cells [47]. In [45], a genetically engineered magneto-sensitive ion channel named Magneto was created. It comprises of a cation ion channel, TRPV4, fused to a paramagnetic protein ferritin. In [46], mechano-sensitive ion channels were controlled via MNPs modulating tension in biological membranes. In [47], MNPs were fused with cilia of hair cells. In all cases, sufficiently strong magnetic fields were required, of the order of 100 mT, i.e., three orders of magnitude larger than $B_{e}$. This is because the corresponding MNPs were either (super)paramagnetic or smaller than 100 nm in linear size, or both. All three above cited papers are already dealing experimentally in fact with artificial magneto-sensitive ion channels or complex nanomagnetic biostructures involving ion channels. In addition, in Ref. [48], a membrane pore forming activity of magnetic nanoparticles has been shown. Hence, such man-made biological structures were already in fact demonstrated, however, for sufficiently strong magnetic fields, much stronger than biological species normally experience.

The idea that a magnetic nanorod can play a role in biological sensing of weak magnetic fields by birds has first been proposed by Yorke [49]. Kirschvink et al. [27,28,50] suggested that it can be a magnetosensitive ion channel that involves a magnetic nanoparticle as sensory element. Several variants of such channels were further proposed and discussed [51,52]. Whether such theoretical proposals can be feasible or not requires, however, a serious detailed investigation of the dynamics of such models [40], which is necessarily stochastic. The sensory element unavoidably experiences friction and random thermal noise caused by the environment, which are related by the fluctuation–dissipation theorem [53,54,55], at a local thermal equilibrium. Cytosol is a viscoelastic liquid, when it is functional, to a first rough approximation, with the main water component which accounts for up to 80% of the cytosol’s mass content. However, it is densely stuffed with different protein polymers, which dramatically enhances the cytosol viscosity for particles of the linear size of 100 nm range and even smaller. Thus, in Refs. [28,29,50], the effective viscosity felt by magnetic nanoparticles is assumed to be 100 times larger than the one of water. Notice that a starving cell can do a transition to an anabiosis state, where cytosol behaves more like a superviscous solid with virtually infinite viscosity [56]. However, we are more interested in its functionally active liquid-like state. The characteristic time scale of sensor was estimated it Refs. [28,50] assuming that the sensor is monostable and it fluctuates around a fixed point, which is not affected by the magnetic field. In that original model, one assumes that the ion channel opens when a critical angle fluctuation occurs, and the amplitude of this fluctuation is affected by the magnetic field. However, most biological ion channels do exhibit a characteristic bistable dynamics while fluctuating between open and closed states [57]. Binhi and Chernavskii considered an orientational bistable dynamics of a magnetosome tethered to cytoskeleton [58,59], however, not in the context of ion channels, but rather stipulating that the above quantum mechanism can be mediated by a fluctuating magnetic field of a magnetosome. Indeed, it can largely exceed one of the Earth [60,61]. Anisotropic field of a spherical ferromagnetic magnetosome is estimated to be up to 402 mT strong near to its surface [61].

The magnetic sensor dynamics of hypothetical ion channels should also be bistable, rather than monostable. Such a model was proposed recently in Ref. [61]. The bistability therein is induced by a gating spring type instability as earlier suggested in the context of hair cell ion channels [62,63]. The analysis of this model for realistic parameters showed, however, that for a viscous friction that is 100 times larger than one in water the time scale of switchings would be so large that such a channel would not be functional. Moreover, the effective friction caused by cytosol for the particles of the size of 100 nm can be even larger, e.g., 1000 fold larger than one in water [64,65,66,67,68,69]. Cytosol as a complex fluid [70,71] is, however, not a Newtonian but rather viscoelastic liquid [72,73,74,75,76,77,78,79,80,81,82,83,84,85], and on the appropriate time scales (probably up to hours in some cases) it is characterized by a slowly, power law decaying memory kernel with a memory cutoff at large times [70,71,86,87]. Integration of this memory kernel yields an effective friction at very large times, when the memory effects can be neglected. The discussed memory friction yields subdiffusion on the relevant time scales, which has been experimentally measured for various nanoparticles in cytosol [56,73,74,77,78,79,82,88,89,90,91,92,93,94,95,96,97,98,99] including magnetosomes [81]. Even smaller particles of the size of only several nanometers can subdiffuse on the time scale up to one hour [74,83]. The non-Markovian dynamics, which includes such effects, has also been studied in Ref. [61] using a Markovian embedding approach of Refs. [87,100]. Contrary to naive reasoning involving a largely enhanced normal viscous friction, however, in accord with the results of non-Markovian rate theory [101,102,103,104,105], it has been shown that such bistable sensors can be functional and operate on a millisecond to second time scale. This is in line with some earlier studies showing that viscoelastic subdiffusion largely accelerates (and not hinders, contrary to a common but misleading interpretation [84]) transport processes in living cells over the naive macroscopic Markovian treatment with a largely enhanced normal viscosity. Viscoelastic power-law memory friction yields non-exponential distribution of the waiting times such as stretched exponential distribution, which elegantly explains [61,87,100] the physical origin of such distributions in ionic channels [106,107,108] and 1/f noise [109], although other approaches also exist [109,110,111,112,113,114,115,116]. In [61], nanosensor rod consists of several nanoparticles. It is coupled by peptide elastic springs to the gating structural elements of several ion channels forming a cluster and can do a large-amplitude orientational motion (about 150 angle degree change), while moving to a metastable state corresponding to the open state of the channels in cluster. In this paper, I will explore the possibility of sensor consisting of the only one sufficiently large nanoparticle and doing a relatively small orientational motion (about 30 degree change) while creating an opening torque on the gates of the channels within a very similar model. It will be shown that such a magnetic sensor is more realistic and it would operate much faster, on biologically relevant time scales, despite a largely enhanced effective friction. Viscoelastic properties of cytosol are very important for this and cannot be disregarded.

## 2. Model

**Figure 1:**
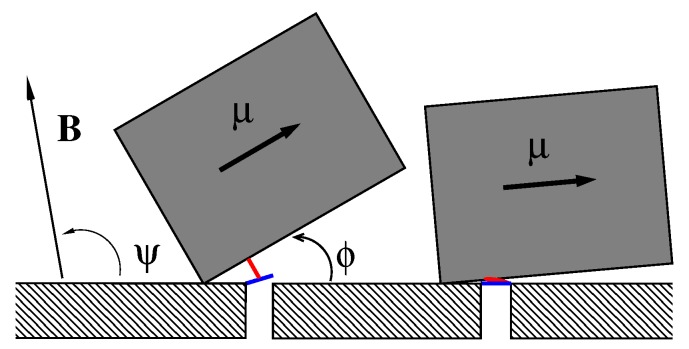
A sketch of the considered model, see the text for detail. Not to scale.

We consider the model sketched in Figure 1, where a single magnetosome consisting of an elongated nanoparticle of magnetite in single-domain ferrimagnetic state of length *L* and width d<L, dressed in a protein–lipid membrane, can rotate around one edge fixed on a cytoskeleton element arming the cell membrane inside the cell. For L=150 nm and d=107 nm its magnetic energy in the magnetic field of the Earth reaches $E_{M}=\mu B_{e}=4.12\cdot 10^{-20}$ J or about $10\;k_{B}T_{r}$ for $T_{r}=297$ K depending on its orientation, which is characterized by the angle ϕ, and the orientation of the magnetic field given by the angle ψ in plane geometry. Biomagnetite nanoparticles of such a size are commonly met both in some bacteria and in the human brain. This “nanocompass” is coupled by *m* elastic peptide linkers (depicted in red) to the gates (depicted in blue) of *m* ionic channels forming a cluster in the membrane (one is depicted). One end of the linker is attached at the distance *l* from the rotation axis to magnetosome and another one to a molecular latch, which forms a gate opening and closing an ion-conducting pore formed by a membrane channel protein. In the absence of magnetic field or for its unfavorable orientation (e.g., ψ=0), the linkers are relaxed (folded) and the channels are closed (right part of Figure 1). For a properly oriented field, the linkers are fully stretched (unfolded) and the channels are open (left part of Figure 1). Even in predominantly closed state channels can stay open time from time due to thermally activated transitions of sensor between its two metastable states depicted in Figure 1. Likewise, in the predominantly open state channels close stochastically in time. The mean time intervals in the states and the mean opening probability of ion channel complex, which determines the ion current controlled by it, strongly depend on the magnetic field. Sensor experiences friction and thermal noise caused by environment, which crucially determine its stochastic dynamics.

**Figure 2:**
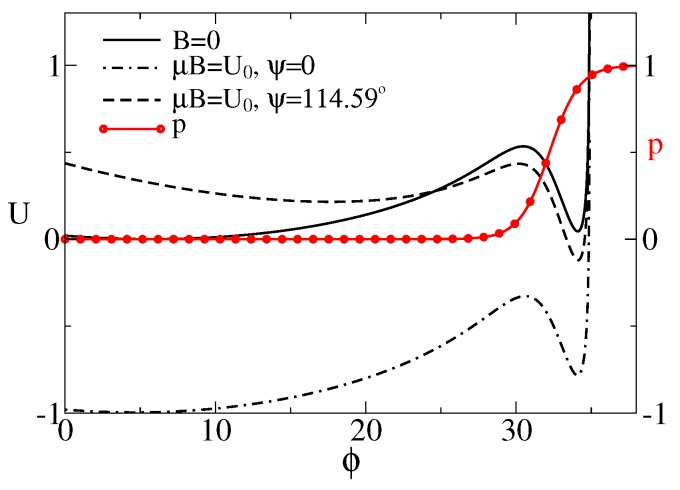
Effective potential for the sensor rotations (in units of $U_{0}$), as a function of its orientation ϕ (in degrees) depending on the presence of magnetic field and its orientation. The motion of sensor is restricted by the plane of membrane and the maximal angle $\phi_{\max}\approx 34.95^{\circ }$ due to the maximal extension length of anharmonic linker. The probability of the cluster to be in the conducting state is also depicted by the red line with symbols. For B=0, or for ψ=0, the global minimum corresponds to the closed state. In the magnetic field of Earth, the cluster is expected to be predominantly in the conducting state, e.g., for $\psi \approx 114.59^{\circ }$ or 2 rad, where the global minimum corresponds to the open state.

The linker, which is an entropic spring provided by a disordered peptide, is modeled by a finite extensible nonlinear elastic (FENE) chain [117]. Its elastic energy depending on the elongation *x* is given by $U_{\mathrm{FENE}}\left(x\right)=-\frac{1}{2}k_{L}l_{\max}^{2}\ln\left(1-x^{2}/l_{\max}^{2}\right)$, where $k_{L}$ is elastic spring constant, and $l_{\max}$ is the maximal extension length of the linker, when it is fully stretched. The rotation of sensor is thus bounded to some angular interval $\left[0,\phi_{\max}\right]$, where $\phi_{\max}$ depends on *l* and $l_{\max}$. We will choose it sufficiently small, like in Figure 1. The channel gate can be in one of two states. The closed state is characterized by the energy $\epsilon_{1}$, and the open one has the energy $\epsilon_{2}-f_{0}x$, which depends on the linker elongation *x*, where $f_{0}$ is a force constant characterizing the strength of coupling (force exerted by the linker on the gate). The gate fluctuates very fast and its dynamics is slaved to a much slower sensor. Statistical mean force exerted by the channel gate on the linker is f(x)=−dG(x)/dx, where $G\left(x\right)=-k_{B}T\ln Z\left(x\right)$, is a corresponding potential of mean force with $Z\left(x\right)=\exp\left[-\beta \epsilon_{1}\right]+\exp\left[-\beta \left(\epsilon_{2}-f_{0}x\right)\right]$ being the statistical sum of the gate, and $\beta =1/(k_{B}T)$ is inverse temperature. The mean force is $f\left(x\right)=f_{0}p\left(x\right)$, where

(1) $$\begin{array}{c} p\left(x\right)=\frac{1}{1+\exp[-f_{0}\left(x-l_{0}\right)/\left(k_{B}T\right)]}, \end{array}$$

is probability of the gate to be open and $l_{0}=\left(\epsilon_{2}-\epsilon_{1}\right)/f_{0}$. To define some $x_{0}$ as equilibrium point, we, following [63], redefine the mean force by a shift as $f\left(x\right)=f_{0}\left[p\left(x\right)-p\left(x_{0}\right)\right]$. Furthermore, the linker elongation is approximated as $x\left(\phi \right)=2l[\sin\left(\phi /2\right)-\sin\left(\phi_{0}/2\right)]$, where $\phi_{0}$ is an equilibrium angle. The potential of mean force or rather torque acting on the rod in our model is [61]

(2) $$\begin{array}{ccc} U(\phi ) & = & -\frac{1}{2}kl_{\max}^{2}\ln\left\{1-{\left[x\left(\phi \right)/l_{\max}\right]}^{2}\right\}\\ & - & k_{B}Tm\ln\left\{1+\exp[f_{0}\left(x\left(\phi \right)-l_{0}\right)/\left(k_{B}T\right)]\right\}\\ & + & mf_{0}p\left(\phi_{0}\right)x\left(\phi \right)-\mu B\cos\left(\psi -\phi \right), \end{array}$$

where $p\left(\phi_{0}\right)=p\left(x=2l\sin\left(\phi_{0}/2\right)\right)$, $k=mk_{L}$. We shall scale the energy in units of $U_{0}=kl_{\max}^{2}$, temperature in the units of $U_{0}/k_{B}$, distances in units of $l_{\max}$, and forces in units of $f_{u}=U_{0}/l_{\max}$. $U_{0}$ will be fixed to $U_{0}=10\;k_{B}T_{r}\approx 41\;\mathrm{pN}\cdot \mathrm{nm}\approx 0.25\;\mathrm{eV}$. For m=7 and a linker with stiffness $k_{L}=0.0429\;\mathrm{pN}/\mathrm{nm}$ [95], k≈0.3pN/nm, this $U_{0}$ corresponds to $l_{\max}\approx 11.69$ nm and force units $f_{u}\approx 3.51$ pN. In this paper, we choose l=2, $l_{0}=0.91$, $f_{0}=3$, $\phi_{0}=0.1\;rad\approx 5.73^{\circ }$. The corresponding U(ϕ) and p(ϕ) are plotted in Figure 2. U(ϕ) is bistable due to a gating spring instability featuring such models [62]. Namely, if the sensor pulls the linker sufficiently strong, another metastable state emerges. Notice that the probability of the channel to be half-open, p=0.5, belongs in this model to the attraction domain of U(ϕ) belonging to the open state. When it becomes lower in energy than one corresponding to the relaxed linker (closed channel), the channel becomes predominantly open. This occurs, e.g., when the magnetic field is applied at the angle $\psi \approx 114.59^{\circ }$.

## 3. Theory and Results

**Figure 3:**
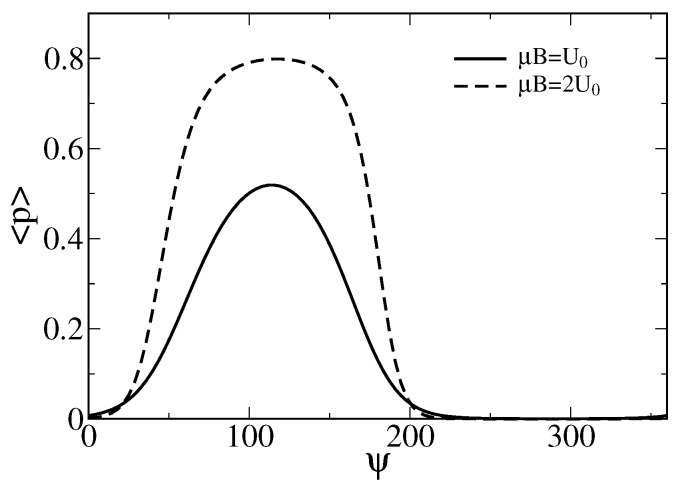
Dependence of the averaged probability 〈p(ψ,B)〉 on the angle ψ (in degrees) for two values of the magnetic energy. One corresponds to $U_{0}=kl_{\max}^{2}$, i.e., a characteristic energy of the stretched gating springs, and another one is double of it. Notice that the sensor operation is possible already for $\mu B_{e}=U_{0}$ with the maximal averaged opening probability over 0.5. For a larger sensor with the linear sizes increased by the factor $2^{1/3}\approx 1.26$, i.e., $189\times 134.8\times 134.8\;{\mathrm{nm}}^{3}$ (also met in living species), the maximal averaged probability increases to about 0.8. Such a sensor would be, however, less sensitive to the variations of ψ near to the maximum.

To obtain the averaged probability 〈p(ψ,B)〉 of the ion channel cluster to be in the open state depending on the strength and orientation of the magnetic field, one has to average p(x(ϕ)) in Equation (1) over ϕ with the statistical weight function $P\left(\psi ,B,\phi \right)=\exp[-U\left(\phi \right)/\left(k_{B}T\right)]/Z$, where $Z=\int_{0}^{\phi_{\max}}\exp\left[-U\left(\phi \right)/\left(k_{B}T\right)\right]d\phi$ is the corresponding statistical sum. The result is shown in Figure 3

The corresponding averaged current through a cluster of ion channel is $\langle I\rangle =mi_{0}\langle p\left(\psi ,B\right)\rangle$, where $i_{0}$ is unitary conductance of a single channel in the cluster. Already single such sensory complex consisting of m=7 large conductance ion channels with $i_{0}∼$50 pA can be sufficient to depolarize the membrane above a sensitivity threshold and evoke spiking activity in hypothetical magneto-sensitive neurons [61].

For the magneto-sensor complex to be functional, its dynamics is, however, also very important. For example, if its characteristic times would lie in the range of minutes or hours, it, for sure, would not be of any relevance for animals.

### 3.1. Stochastic Dynamics without Memory

**Figure 4:**
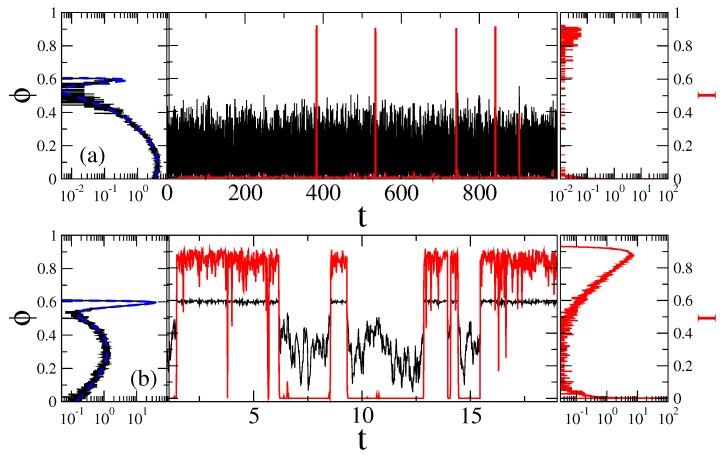
Sample trajectories (central part) and the distributions of the sensor orientation (left part), as well as distribution of current values (right part) for $\mu B=U_{0}$ at two fixed magnetic field angles: (**a**) ψ=0, with ionic channels being predominantly closed; and (**b**) ψ = 2 rad $\approx 114.59^{\circ }$, where channels are predominantly open, in the case of Markovian memoryless dynamics. Black curves correspond to the motion of sensor and the red ones to fluctuations of ionic current due to open-closed gating dynamics. The dashed blue lines in the left parts are the corresponding theoretical values of distribution, $P\left(\psi ,B,\phi \right)=\exp[-U\left(\phi \right)/\left(k_{B}T\right)]/Z$, with U(ϕ) in Figure 2. Time is in units of $\tau_{sc}=0.17$ ms, angle in radians, and current in the units of a maximal current possible.

First, we consider stochastic orientational dynamics of the sensor in the viscous medium under the mean torque f(ϕ)=−∂U(∂)/∂ϕ, viscous friction term $F_{v}=-\eta_{0}\dot{\phi }$, with orientational friction coefficient $\eta_{0},$ and the corresponding white Gaussian thermal noise $\xi_{0}\left(t\right)$ of the environment. The last two are related by the fluctuation–dissipation relation (FDR) named also the second fluctuation–dissipation theorem (FDT) by Kubo [53,54,55], $\langle \xi_{0}\left(t^{\prime }\right)\xi_{0}\left(t\right)=2k_{B}T\eta_{0}\delta \left(t-t^{\prime }\right)$, at the medium’s temperature *T*. δ(t) here is the Dirac’s delta function signaling that this noise has an infinite root-mean squared amplitude—a common singular model in statistical physics. The overdamped stochastic dynamics reads [54,55,118]

(3) $$\begin{array}{c} \eta_{0}\dot{\phi }=f\left(\phi \right)+\xi_{0}\left(t\right)\;, \end{array}$$

and a characteristic time-scale entering it is $\tau_{sc}=\eta_{0}/U_{0}$. Precise estimation of the rotational frictional coefficient for the particle of the form considered is not easy [119]. A simplest estimate can be obtained by replacing it with the sphere of equal volume $V=Ld^{2}$. Then, $\eta_{0}∼$6$\zeta_{0}V$, where $\zeta_{0}$ is the medium’s viscosity. For water at *T* = 20 ${}^{\circ }$C with $\zeta_{0}∼$ 1 mPa·s, we obtain $\tau_{sc}∼$ 0.17 ms, which is much smaller than for the rod-like sensor in [61]. This is a first reason why it is faster. $\tau_{sc}$ is the time scale used in our simulations done using the second-order stochastic Runge–Kutta method, or stochastic Heun method [120], see in Methods. The sample trajectories are shown in Figure 4 both for predominantly closed channels (part a, for ψ=0), and for predominantly open channels (part b, ψ=2 rad.)

From very long single trajectories we extract residence time distributions in open and closed states using the following procedure. Two thresholds are placed at the potential minima of the sensor metastable states corresponding to U(ϕ) or, equivalently, two maxima of the probability distribution of the ionic current. The residence time interval in the closed state starts after a first downward crossing of the lower threshold and continues until the first upward crossing of the higher threshold. Likewise, the residence time interval in the open state starts after a first upward crossing of the upper threshold and continues until the first downward crossing of the lower threshold. This allows to map the continuous fluctuation processes in Figure 4 (central part) onto the corresponding two-state, on-off processes, characterized by the survival probabilities of the residence time-intervals in the corresponding states, $P_{c}\left(\tau \right)$ and $P_{o}\left(\tau \right)$. In the case of sufficiently large potential barriers, the Markovian continuous state dynamics yields also two-state Markovian dynamics completely characterized by the exponential survival probabilities $P_{c,o}\left(\tau \right)=\exp\left(-r_{o,c}\tau \right)$, and the corresponding probability densities $\psi_{c,o}\left(\tau \right)=-dP_{c,o}\left(\tau \right)/d\tau =r_{o,c}\exp\left(-r_{o,c}\tau \right)$, where $r_{o}=1/\langle \tau_{c}\rangle$, and $r_{c}=1/\langle \tau_{o}\rangle$ are the opening and closing rates, respectively. In the case of Markovian two-state dynamics, they are inverse of the mean residence times in the closed state, $\langle \tau_{c}\rangle$, and the open state, $\langle \tau_{o}\rangle$, correspondingly. As a more general model, we use a stretched-exponential or Weibull distribution

(4) $$\begin{array}{c} P_{c,o}\left(\tau \right)=c_{c,o}\exp\left[-{\left(\tau /\tau_{c,o}\right)}^{\beta_{c,o}}\right] \end{array}$$

for the whole survival probability derived from numerics (then, $c_{c,o}=1$) or some parts of it. The corresponding density

(5) $$\begin{array}{c} \psi_{c,o}\left(\tau \right)=\frac{c_{c,o}\beta_{c,o}}{\tau_{c,o}^{\beta_{c,o}}}\frac{1}{\tau^{1-\beta_{c,o}}}\exp\left[-{\left(\tau /\tau_{c,o}\right)}^{\beta_{c,o}}\right] \end{array}$$

has a decaying power law part $1/\tau^{1-\beta_{c,o}}$, for $0<\beta_{c,o}<1$ which is the reason why this distribution can be confused for a truly power-law dependence on the corresponding plots for a large $\tau_{c,o}$. To avoid this pitfall of interpretation, the plots of survival probability P(τ) can be preferred.

**Figure 5:**
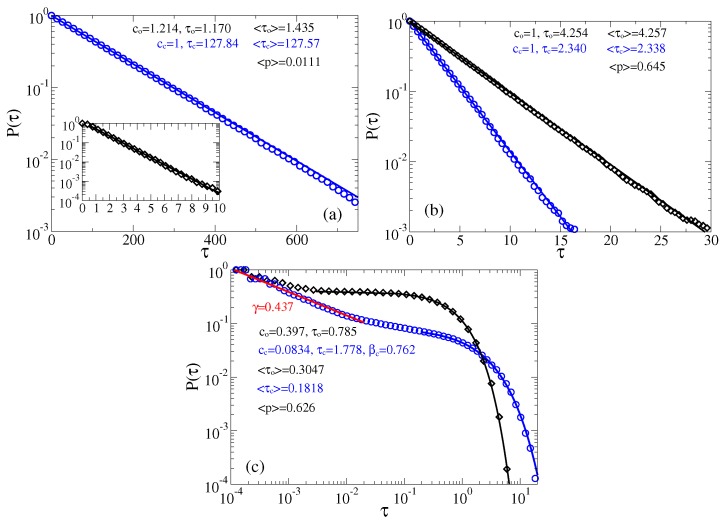
Survival probabilities of open and closed times in the case of memoryless dynamics for the channels: (**a**) predominantly closed, $\mu B=U_{0}$, ψ=0; and (**b**,**c**) predominantly open, $\mu B=U_{0}$, ψ=2. (**a**,**b**) The residence time distributions are extracted by placing two thresholds at the sensor orientations corresponding to the maxima of probability distribution of the sensor orientations (the left panel in Figure 4), or, equivalently, the current values (the right panel in Figure 4). (**c**) Only one threshold is used at the minimum of current distribution corresponding to p=0.2 and the top of U(ϕ) barrier separating two metastable states. Notice, that many re-crossings of this threshold occur when the sensor dwells on the top of this barrier. A measuring device with a finite time resolution $\Delta t_{res}$ will miss many of such events. We model this by using $\Delta t_{res}=100\delta t$, where $\delta t=2\cdot 10^{-6}$ is the time step in simulations. Notice that this *incorrect* procedure leads to spurious power law and stretched exponential distributions with the parameters shown in the plot. It results also in far too small values of the mean residence times, $\langle \tau_{c}\rangle$, $\langle \tau_{o}\rangle$, as compare with the correct values in the part (**b**). Clearly, these “measurable” mean times will become even much smaller for $\Delta t_{res}=\delta t$. This procedure with one threshold placed at top of the barrier separating two basins of attraction is hence very subjective and it cannot be trusted.

The just described procedure of extracting two-state process is well known (see, e.g., in [61,100]). In [61], the derived in this way exponential distributions agree very well with the results of Kramers theory for the transition rates [105], which confirms that this procedure is essentially correct. In the present case, the potential barriers are smaller and the curvatures of the potential wells (at the bottom), and the barrier (at the top) are very different. In such a situation, a good agreement with the Kramers theory is not expected. In this respect, notice large fluctuations of the sensor orientation in Figure 4 in the case of the relaxed linker and the closed state of channel. In a sharp contrast, these fluctuations are much smaller when the linker is in its tense state. This does not mean, however, that there cannot be large fluctuations of conductance when the linker is tense. Vice versa, they are much larger when the linker is tense than when it is relaxed. This is because the midpoint of p(ϕ) dependence belongs to the attraction basin of the metastable state of U(ϕ) that corresponds to the open state, and the barrier corresponds to p(ϕ)≈0.2 (see Figure 2). The amplitude of current fluctuations which corresponds the large-amplitude fluctuations of sensor with the relaxed linker is quite small because of an exponential dependence in Equation (1). The numerics for the case of predominantly closed channels in Figure 5a show that the closed time residence time distribution is nearly exponential $c_{c}=1$, and $\tau_{c}\approx \langle \tau_{c}\rangle$. However, the open time distribution is not quite exponential. Initially, it is different from exponential, see in inset of Figure 5a, which is the reason why $c_{o}\approx 1.214>1$ and fitted $\tau_{o}$ does not agree well with $\langle \tau_{o}\rangle$. The corresponding mean opening probability is calculated as $\langle p\rangle =\langle \tau_{o}\rangle /\left(\langle \tau_{o}\rangle +\langle \tau_{c}\rangle \right)$, and it is quite small in Figure 5a. For predominantly open channels in Figure 5b, both open and closed time distributions are nearly exponential. The corresponding mean times $\langle \tau_{o}\rangle =4.254\times 0.17\approx 0.723$ ms and $\langle \tau_{o}\rangle =2.338\times 0.17\approx 0.397$ ms are quite small, being typical for very fast ion channels like sodium channels, which are crucial for neuronal excitability. Notice also that the corresponding 〈p〉≈0.645 is somewhat larger than 〈p〉 calculated for the continuous process in Figure 2.

#### Separation of Closed and Open States with a Single Threshold

The distribution of current values in the right panel of Figure 4 implies, however, that it might be difficult practically implement the detecting procedure with two thresholds because the lower threshold must be set at a very tiny, $10^{-8}-10^{-6}$, value of current. What a theorist can easily do in a *Gedankenexperiment*, an experimentalist can have difficulties to realize in practice. Setting the lower threshold at some other higher value, say 0.2, can very essentially modify the thus derived statistics [61], and, hence, it is a rather arbitrary procedure. Another common procedure, which some experimentalists implement both in ion channel research [57], and, especially, in deriving statistics of blinking quantum dots [121,122,123,124] is to operate with the only one detection threshold. It is “naturally” placed at the minimum of the current distribution separating two maxima. An immediate objection of a theorist experienced in the rate theory is that this procedure corresponds to placing a separation threshold on the top of potential barrier (for the sensor, in our case) separating two metastable basins of attraction. It might first look natural. However, physical picture of the rate transitions between two metastable basins of attraction says that it is not. In fact, the particle dwells mostly in a potential well and seldomly, with rate $r_{0}=\left(\omega_{0}/2\pi \right)\exp\left[-\Delta U/\left(k_{B}T\right)\right]$, where $\omega_{0}$ is circular frequency of oscillations near the bottom of the potential well, and ΔU is the height of the potential barrier, comes on the barrier top. However, it generally can dwell for a while on the top of this potential barrier and re-cross this single threshold many times, before the particle will make finally transition to another potential well. These multiple crossings yield the so-called transmission coefficient which being multiplied with $r_{0}$ renders the resulting rate much smaller. The whole rate theory is, roughly speaking, about how to calculate this transmission coefficient, which depends on friction, etc. Its maximal value is one (single crossing). This is why for the overdamped dynamics considered, this second procedure yields totally different residence time distributions, which severally distort the correct distributions characterizing two-state dynamics. In particular, the mean residence times derived in such a way will be much smaller than the correct ones. Experimentally, the problem is softened and can be masked by a finite time resolution $\Delta t_{res}$ of a measurement device. This is why not every fast recrossing will be measured. With $\Delta t_{res}$ becoming large many such recrossing events will be missed. However, this does not mean that statistics will become closer to the correct one. Not at all. To model this second procedure we use $\Delta t_{res}=100\delta t$, where δt is the time step used in simulations, and the corresponding results are depicted in Figure 5c. First, the mean residence times are indeed much smaller (they became naturally even much more smaller for $\Delta t_{res}=\delta t$). Second, the residence time statistics is severely distort. In particular, about 90% of the closed time intervals follow now a spurious power law, $P_{c}\left(\tau \right)\propto 1/\tau^{\gamma }$, with γ≈0.437, and hence $\psi_{c}\left(\tau \right)\propto 1/\tau^{1+\gamma }=1/\tau^{1.437}$. Similar power laws are indeed measured for quantum dots using a similar approach with one threshold (however, the physics there is very different and our reasoning cannot be directly applied). Moreover, a spurious stretched exponential tail appears with $\beta_{c}=0.762$ and weight $c_{c}=0.0834$. The open time distribution has, however, an exponential tail, $\beta_{o}=1$. Our example shows explicitly how dangerous can be this second detection method and why it cannot be trusted. Interestingly, this second procedure barely affects 〈p〉, cf. 〈p〉=0.642 in Figure 5b vs. 〈p〉=0.626 in Figure 5c.

Notice that with a naíve replacement $\tau_{sc}\to 100\tau_{sc}=17$ ms in a medium with effective viscosity 100× larger than one of water our sensor dynamics would become 100× slower what would essentially deteriorate its functionality. A much slower model channel in [61] would even cease to be of any interest in biological context, if to apply this Markovian reasoning with $\eta_{\mathrm{eff}}$ = 100 $\eta_{0}$. This, however, does not happen in fact in viscoelastic media such as cytosol, where a more careful treatment is required, since the viscoelastic memory effects become very essential.

### 3.2. Stochastic Dynamics in Viscoelastic Environment

In viscoelastic crowded environments such as cytosol, apart from viscous friction caused by the its main water component, also a viscoelastic memory friction is present, $F_{v-el}\left(t\right)=\int_{0}^{t}\eta_{\mathrm{mem}}\left(t-t^{\prime }\right)\dot{\phi }\left(t^{\prime }\right)dt^{\prime }$, where $\eta_{\mathrm{mem}}\left(t\right)$ is a memory kernel. It is necessarily complemented by a corresponding correlated thermal unbiased Gaussian random force of the environment, $\xi_{\mathrm{mem}}\left(t\right)$. In accordance with the (second) FDT, $\langle \xi_{\mathrm{mem}}\left(t\right)\xi_{\mathrm{mem}}\left(t^{\prime }\right)\rangle =k_{B}T\eta_{\mathrm{mem}}(|t-t^{\prime }\left|\right)$. Sensor dynamics in this case obeys a generalized Langevin equation (GLE) reading

(6) $$\begin{array}{c} \eta_{0}\dot{\phi }=f\left(\phi \right)-\int_{0}^{t}\eta_{\mathrm{mem}}\left(t-t^{\prime }\right)\dot{\phi }\left(t^{\prime }\right)dt^{\prime }+\xi_{\mathrm{mem}}\left(t\right)+\xi_{0}\left(t\right)\;. \end{array}$$

**Figure 6:**
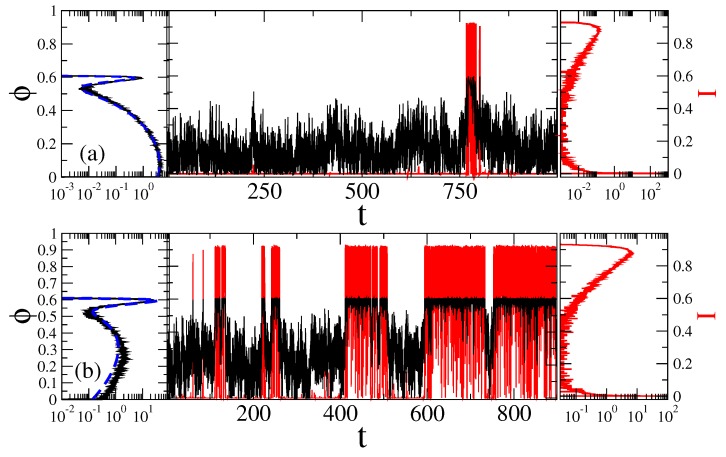
Sample trajectories (central part) and the distributions of the sensor orientation (left part), as well as distributions of the current values (right part) for $\mu B=U_{0}$ at two fixed magnetic field angles: (**a**) ψ=0, with ionic channels being predominantly closed; and (**b**) ψ = 2 rad $\approx 114.59^{\circ }$, where channels are predominantly open, in the case of non-Markovian fractional dynamics with $\eta_{\mathrm{eff}}=1000$, $\eta_{\alpha }\approx 10$ $\eta_{0}$. Black curves correspond to the motion of sensor and the red ones to fluctuations of ionic current due to open-closed gating dynamics. The dashed blue lines in the left parts are the corresponding theoretical values of distribution, $P\left(\psi ,B,\phi \right)=\exp[-U\left(\phi \right)/\left(k_{B}T\right)]/Z$, with U(ϕ) in Figure 2. Time is in units of $\tau_{sc}=0.17$ ms, angle in radians, and current in the units of a maximal current possible.

The simplest Maxwellian model of viscoelasticity corresponds to an exponentially decaying memory kernel, $\eta_{\mathrm{mem}}=k_{1}\exp\left(-\nu_{1}t\right)$, where $k_{1}$ is a spring constant and $\nu_{1}$ is a relaxation rate of stress. For $\nu_{1}\to 0$, $F_{v-el}\left(t\right)$ behaves as an elastic force, while in the limit $k_{1}\to \infty$, $\nu_{1}\to \infty$, $\eta_{1}=k_{1}/\eta_{1}=const$ it corresponds to the viscous Stokes friction with the friction coefficient $\eta_{1}$. This is how Maxwell derived the phenomenon of viscosity from the phenomenon of elasticity, i.e., by letting the elastic stress to relax in time [87]. Complex viscoelastic liquids and gels are, however, characterized by a power law decaying memory kernel, $\eta_{\mathrm{mem}}\left(t\right)=\eta_{\alpha }t^{-\alpha }/\Gamma \left(1-\alpha \right)$, with 0<α<1, as it has first been established by Gemant [87,100,125], and which now presents a common model. In this particular case, $F_{v-el}\left(t\right)$ can be abbreviated as $F_{v-el}\left(t\right)=\eta_{\alpha }d^{\alpha }\phi \left(t\right)/dt^{\alpha }$, which just defines the fractional Caputo derivative of the order α. Hence, $\eta_{\alpha }$ is customarily named the fractional friction coefficient, and GLE in this particular case is named fractional Langevin equation or FLE. This is, of course, an idealization. In reality, there are always two memory cutoffs present. A large time memory cutoff $\tau_{h}=1/\nu_{l}$ defines the slowest Maxwellian relaxation mode of the environment, and with $\eta_{\alpha }\to \eta_{\alpha }\exp\left(-\nu_{h}t\right)$, an effective friction coefficient $\eta_{\mathrm{eff}}=\int_{0}^{\infty }\eta_{\mathrm{mem}}\left(t\right)dt=\eta_{\alpha }\tau_{h}^{1-\alpha }$ can be introduced. Notice that $\eta_{\mathrm{eff}}$ characterizes diffusion on the time scale $t\gg \tau_{h}$. However, $\tau_{h}$ can be well in the range of minutes and even hours. It depends on the system considered. We assume it to be in the range of seconds for our nanosensor. As long as $t<\tau_{h}$, e.g., for the duration of sojourn times of our sensor in the metastable states, it is $\eta_{\alpha }$ that determines the stochastic dynamics and not $\eta_{\mathrm{eff}}$, which can be effectively infinite, with $\tau_{h}\to \infty$. This is the reason why thinking in terms of some $\eta_{\mathrm{eff}}$ can be very misleading for viscoelastic media. This is a macroscopic type approximation, which can fail completely on micro- and nano-scales. With this reservation we use it because far too many researchers continue to think in terms of some $\eta_{\mathrm{eff}}$. In the simulations presented below we fixed α=0.5 (one of common experimental values for cytosol [74]) and $\tau_{h}=10^{4}$ (or about 1.7 s, when $\tau_{sc}=0.17$ ms). $\eta_{\alpha }$ will be fixed to two values by choosing $\eta_{\mathrm{eff}}$ = 100 $\eta_{0}$ and $\eta_{\mathrm{eff}}$ = 1000 $\eta_{0}$. In dimensionless units used in numerics, the former (intermediate) fractional friction is about $\eta_{\alpha }\approx \eta_{0}$, whereas the latter one (strong) is $\eta_{\alpha }\approx 10$ $\eta_{0}$. For intermediate $\eta_{\alpha }$, the relaxation within a potential well is mostly exponential with a heavy power law tail, while for the large $\eta_{\alpha }$, it is initially stretched exponential and then changes into a power-law decay (Mittag-Leffler relaxation function) [61]. This latter one corresponds to dielectric Cole–Cole response [126] which is typical for biological media [4]. Furthermore, on physical grounds also a short time cutoff $\tau_{l}=1/\nu_{h}$ is always necessarily present. It ensures that spectral density of the noise $\xi_{\mathrm{mem}}\left(t\right)$ does not contain frequencies much above $\nu_{h}$. This physically corrects the approximation of continuum medium where such a cutoff is absent because this approximation neglects the atomistic nature of any real condensed medium. In our numerics, we take it to be $\nu_{h}=\nu_{0}=10^{4}$, and the numerical method is based on approximating the memory kernel by a sum of exponentials, and using a Markovian embedding (9) of the GLE dynamics in Equation (6), see in Methods. This allows for a numerically highly accurate approach, with a well controlled accuracy [87,100]. Typical trajectories for strong fractional friction are shown in Figure 6. For a weak or intermediate fractional friction, they look more like the ones in Figure 4. One striking feature is immediately seen in Figure 6. This is a highly bursting character of sojourns in the open state, where huge many very short excursions happen to the closed state during a long sojourn in the open state. It visually signals a truly non-exponential kinetics.

#### 3.2.1. Intermediate fractional friction, $\eta_{\alpha }\approx \eta_{0}$

**Figure 7:**
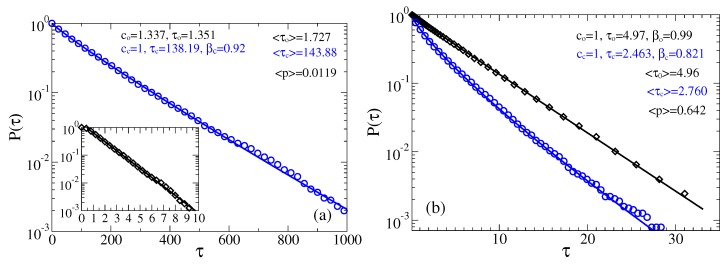
Survival probabilities of open and closed times in the case of non-Markovian fractional dynamics with $\eta_{\mathrm{eff}}=100$, $\eta_{\alpha }\approx \eta_{0}$: (**a**) $\mu B=U_{0}$ and ψ=0; and (**b**) $\mu B=U_{0}$ and ψ=2. The residence time distributions are extracted by placing two thresholds at the sensor orientations corresponding to the maxima of probability distribution.

The first profound influence of fractional viscoelastic friction on the statistics of the residence time distributions can be reveal in Figure 7. Namely, the distribution of closed times becomes stretched exponential with $\beta_{c}\approx 0.92$ in part a, and $\beta_{c}\approx 0.82$ in part b. However, the distribution of open times remains almost exponential, and the mean times in the states and the mean opening probability remain only weakly affected, compare with Figure 5a,b. This is especially striking because we can further arbitrarily increase $\eta_{\mathrm{eff}}$, while keeping the same $\eta_{\alpha }$. This is easy to do in our numerics just by further increasing the number *N* of exponentials in Equation (7) and, correspondingly, the Markovian embedding dimension in Equation (9). The results will not be changed because the essential kinetics in Figure 7 is on the time scale which is already smaller than the cutoff time $\tau_{h}$, that further increases with *N*. This feature must be shocking for all those who continue thinking in terms of $\eta_{\mathrm{eff}}$, rather than fractional friction featuring such complex media as cytosol. All in all, for such an intermediate $\eta_{\alpha }$, our sensor operates as fast as in water. This is a good news.

#### 3.2.2. Strong Fractional Friction, $\eta_{\alpha }\approx$ 10 $\eta_{0}$

**Figure 8:**
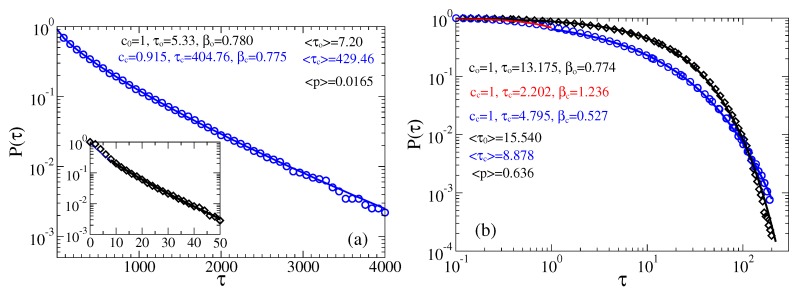
Survival probabilities of open and closed times in the case of non-Markovian fractional dynamics with $\eta_{\mathrm{eff}}=1000$, $\eta_{\alpha }\approx 10\eta_{0}$: (**a**) $\mu B=U_{0}$ and ψ=0; and (**b**) $\mu B=U_{0}$ and ψ=2. The residence time distributions are extracted by placing two thresholds at the sensor orientations corresponding to the maxima of probability distribution.

For a strong fractional friction, both closed and open times have Weibull distribution with smaller values of the stretched exponential parameter β (see Figure 8). Here, the influence of viscoelastic effects is very strong. Initially, for small time, β can exceed one, see for closed times (red fitting curve) in part b. However, the mean opening probabilities are also almost unaffected, as compared with the Markovian case, despite the mean residence times increase. This increase is not very strong, by a factor of less than three only. Our sensor remains very fast.

## 4. Discussion

Design of a magnetosensitive ion channel complex using a single biomagnetite nanoparticle as a magnetic field sensor makes it more realistic. In essence, the model studied in this paper is a variant of the model in Ref. [61]. The differences are in detail. However, these details do matter. First, a single prolonged nanoparticle is used instead of a rod made of 5÷7 such nanoparticles, and, second, a shorter linker is used to restrict the orientational motion of sensor when it fluctuates between two metastable states in response to a change of the magnetic field orientation. In our present work, it is just about $30^{\circ }$, whereas, in [61], it is about $150^{\circ }$. This makes the present variant much faster and more realistic in view of possible sterical restrictions within the cell. This comes, however, at a price: the magnetic moment of the sensor must be larger to ensure its magnetic energy to be about $10\;k_{B}T$ in the magnetic fields of Earth vs. $3÷4\;k_{B}T$ in Ref. [61]. This is, however, not a problem because such sufficiently large nanoparticles are met both in certain bacteria, and in the human brain, even if they are certainly less common that ones assumed in [61]. Furthermore, our analysis shows that such a sensor would be operating very fast even in viscoelastic cytosol feeling an effective viscosity 1000× larger than one of water. More precisely, this effective viscosity can be even formally infinite, like in solid, because this is not an effective macroscopic friction defined on a very large time scale which determines the stochastic switching dynamics. In fact, the microscopic fractional friction does matter here and it is crucially relevant. If it is large and dominates the dynamics, as in the studied case of $\eta_{\mathrm{eff}}$ = 1000 $\eta_{0}$, the “off–on” two-state dynamics becomes very bursting. It is not characterized by nearly exponential distributions of the residence times in two states anymore, but rather by profoundly non-exponential stretched exponential distributions. Such distributions are indeed measured in several biological ion channels, and our theory tentatively explains their principal origin as one rooted in the viscoelasticity of environment. All this clearly correlates with the dielectric Cole–Cole response in such media (Mittag-Leffler viscoelastic relaxation). It is important that the mean residence times (MRTs) as well as all higher moments remain finite; moreover, MRTs were increased in our model study by a factor of less than three only, as compared to ones in water. Arguably, it can be unbelievable and embarrassing for those who continue to think in terms of an $\eta_{0}\to \eta_{\mathrm{eff}}$ renormalization within a Markovian dynamics. However, this is a result of the proper treatment of non-Markovian effects, and it presents a very good news with respect to feasibility of such magnetosensitive complexes in living systems.

If such magnetosensitive ion channels do exist, why then they were not found until now? The situation here can be similar to ion channels associated with cilia in hair cells. The existence of those channels is widely assumed and is taken nowadays by many almost as a real fact. However, they were not identified until now as biological protein structures, such as many other well-known ion channels, despite numerous efforts. It is very difficult to identify them because each cilia is assumed to be connected by elastic protein linkers to the gates of many such channels, and it is difficult to confirm this hypothesis experimentally. The ion channels of cilia in hair cells remain elusive, even if in their existence practically nobody doubts. The hypothesis of magnetosensitive ion channel complexes is much less studied. However, it is a reasonable one and it should attract more attention in the future.

## 5. Methods

The power law memory kernel is approximated between two cutoffs by a sum of exponentials

(7) $$\begin{array}{c} \eta_{\mathrm{mem}}\left(t\right)=\sum_{i=1}^{N}k_{i}\exp\left(-\nu_{i}t\right), \end{array}$$

with a fractal scaling of relaxation rates $\nu_{i}=\nu_{0}/b^{i-1}$ and weights $k_{i}\propto \nu_{i}^{\alpha }$. Namely,

(8) $$\begin{array}{c} k_{i}=\nu_{0}\eta_{\mathrm{eff}}\frac{b^{1-\alpha }-1}{b^{(i-1)\alpha }\left[b^{N(1-\alpha )}-1\right]}. \end{array}$$

Here, $\nu_{0}=\nu_{h}=1/\tau_{l}$ is the largest viscoelastic rate of the environment. Using scaling or dilation parameter b=10 allows to achieve 4% accuracy of power law approximation between two cutoffs for α=0.5 and a sufficiently large *N* [87,100]. In our numerics, we use $\nu_{0}=10^{4}$ and N=9, $\tau_{h}=b^{N-1}/\nu_{0}=10^{4}$. The fractional friction coefficient is $\eta_{\alpha }=\eta_{\mathrm{eff}}\tau_{h}^{\alpha -1}/g_{\alpha }$, with an inverse proportionality coefficient $g_{\alpha }$, which slightly differs from unity. For α=0.5, b=10, and N>5, $g_{\alpha }\approx 1.07$. This allows for a Markovian embedding

(9) $$\begin{array}{ccc} \eta_{0}\dot{\phi } & = & f\left(\phi \right)-\sum_{i=1}^{N}k_{i}\left(\phi -y_{i}\right)+\xi_{0}\left(t\right),\\ \eta_{i}\dot{y_{i}} & = & k_{i}\left(\phi -y_{i}\right)+\xi_{i}\left(t\right), \end{array}$$

of GLE dynamics (6) in a space of N+1 dimension. Here, $y_{i}$ are non-dimensional linear auxiliary variables, $\eta_{i}=k_{i}/\nu_{i}$, and $\xi_{i}\left(t\right)$ are mutually independent auxiliary uncorrelated white Gaussian noises,

(10) $$\begin{array}{ccc} \langle \xi_{i}\left(t\right)\xi_{j}\left(t^{\prime }\right)\rangle & = & 2\delta_{ij}k_{B}T\eta_{j}\delta \left(t-t^{\prime }\right). \end{array}$$

They are also uncorrelated with $\xi_{0}\left(t\right)$. The initial angles $y_{i}\left(0\right)$ are sampled from Gaussian distributions centered around ϕ(0), $\langle y_{i}\left(0\right)\rangle =\phi \left(0\right)$ with variances $\langle {\left[y_{i}\left(0\right)-\phi \left(0\right)\right]}^{2}\rangle =k_{B}T/k_{i}$, in order to have a complete equivalence with the corresponding GLE in Equations (6) and (7) in the ensemble sense [87]. The system of stochastic differential Equations (9) is propagated in time (in units of $\tau_{sc}=\eta_{0}/U_{0}$) using stochastic Heun method [120]. The time step was fixed to $\delta t=2\cdot 10^{-6}$. $T=T_{r}=297$ K, while $U_{0}=10\;k_{B}T_{r}$.

## 6. Conclusions

To conclude, in this paper, we studied a model of magnetosensitive ion channel complexes in more realistic detail. Our study underpins theoretically a possible existence of such complexes which should attract more attention of researchers trying to resolve the puzzle of magnetosensitivity of many animals to environmental magnetic fields. The author is convinced that ion channels are involved in magnetosensing, even if the concrete structures involving them can be different. More research in this direction is required and welcome.
